# Metabolomics: a promising tool for deciphering metabolic impairment in heavy metal toxicities

**DOI:** 10.3389/fmolb.2023.1218497

**Published:** 2023-07-06

**Authors:** Muhammad Sajid Hamid Akash, Azka Yaqoob, Kanwal Rehman, Muhammad Imran, Mohammed A. Assiri, Fatema Al-Rashed, Fahd Al-Mulla, Rasheed Ahmad, Sardar Sindhu

**Affiliations:** ^1^ Department of Pharmaceutical Chemistry, Government College University Faisalabad, Faisalabad, Pakistan; ^2^ Department of Pharmacy, The Women University, Multan, Pakistan; ^3^ Research Center for Advanced Materials Science (RCAMS), King Khalid University, Abha, Saudi Arabia; ^4^ Department of Chemistry, Faculty of Science, King Khalid University, Abha, Saudi Arabia; ^5^ Immunology and Microbiology Department, Dasman Diabetes Institute, Dasman, Kuwait; ^6^ Research Division, Dasman Diabetes Institute, Dasman, Kuwait; ^7^ Animal and Imaging Core Facilities, Dasman Diabetes Institute, Dasman, Kuwait

**Keywords:** heavy metals, toxicity, metabolomics, metabolic impairment, oxidative stress

## Abstract

Heavy metals are the metal compounds found in earth’s crust and have densities higher than that of water. Common heavy metals include the lead, arsenic, mercury, cadmium, copper, manganese, chromium, nickel, and aluminum. Their environmental levels are consistently rising above the permissible limits and they are highly toxic as enter living systems via inhalation, ingestion, or inoculation. Prolonged exposures cause the disruption of metabolism, altered gene and/or protein expression, and dysregulated metabolite profiles. Metabolomics is a state of the art analytical tool widely used for pathomolecular inv22estigations, biomarkers, drug discovery and validation of biotransformation pathways in the fields of biomedicine, nutrition, agriculture, and industry. Here, we overview studies using metabolomics as a dynamic tool to decipher the mechanisms of metabolic impairment related to heavy metal toxicities caused by the environmental or experimental exposures in different living systems. These investigations highlight the key role of metabolomics in identifying perturbations in pathways of lipid and amino acid metabolism, with a critical role of oxidative stress in metabolic impairment. We present the conclusions with future perspectives on metabolomics applications in meeting emerging needs.

## 1 Introduction

The profiling of metabolome is known as metabonomics or metabolomics, which is a branch of “omics” science including genomics, proteomics, transcriptomics, inomics, phenomics, and metabolomics (Deshmukh et al., 2014). Metabonomics has been frequently associated with nuclear magnetic resonance (NMR) spectroscopy while metabolomics is often associated with mass spectrometry (MS) based approaches and refers to the study of metabolites, metabolite composition, metabolome, or metabolism in the organism, organ, tissue, or cell. It includes the quantitative and qualitative characterization of small molecule (<1.5 kDa) metabolites, their intermediates, hormones, and other signaling molecules as well as secondary metabolites that show changes in response to genetic modifications or stimuli from the external and/or internal sources. The metabolome represents the global collection or complete set of all low-molecular weight metabolites that are expressed during the metabolic activity and provide a direct functional readout of the cellular activity and the physiological status (Patti et al., 2012; Dinis-Oliveira, 2014; Wishart, 2016).

Metabolomics is located at the bottom of the “omics” cascade and relates to both the downstream output of the genome and the upstream input of the environment and may, therefore, answers queries that might not be answered using other branches of “omics” science (Kettunen et al., 2012; Wishart, 2016; Zaitsu et al., 2016). Whereas, metabolome refers to the metabolites and biomolecules derived endogenously including steroids, fatty acids, lipids, vitamins, carbohydrates, and amino acids, the exo-metabolome refers to the extracellular metabolites and the xeno-metabolome relates to the metabolites derived exogenously from xenobiotics or their metabolites from phase I or phase II metabolism (David et al., 2014). Metabolomics helps detect endogenous substances in biospecimens such as tissues, hair, nails, blood, urine, cerebrospinal fluid, saliva, etc. in relation to changes in metabolite profiles under different conditions. Metabolomics is now being widely used in studies of biomarkers (Klein and Shearer, 2016; Zhang et al., 2016; Ambati et al., 2017), drug discovery (Lu and Chen, 2017; Mercier et al., 2018), biotransformation pathways validation (Ren et al., 2016; Zhang et al., 2016), disease pathogenesis (Klein and Shearer, 2016; Ren et al., 2016; Würtz et al., 2016), and the investigations related to toxicology, nutrition, pharmacology, and clinical trials (Brignardello et al., 2017; Korsholm et al., 2017; Cornelis et al., 2018; Wu et al., 2018).

The metals found in earth’s crust with density higher than that of water are called heavy metals (Tchounwou et al., 2012). Humans and animals are constantly being exposed to heavy metals and, unfortunately, their levels in the environment are exceeding the permissible limits. Contamination of heavy metals in water resources, air, and food is a metter of growing concern for both human and animal health and wellbeing, and hundreds of amillions of people are being affected worldwide (Balali-Mood et al., 2021). The levels of toxic heavy metals in the environment have reached the alarming thresholds, posing a serious global issue due to their potential adverse effects on living systems. These inorganic pollutants, commonly known as heavy metals, are continuously increasing in our surroundings as they are released into the atmosphere, soil, and water bodies. The primary sources of these heavy metals include the rapid expansion of metal industries, agricultural practices, the use of fertilizers, improper waste disposal, and the widespread application of pesticides (Rajkumar et al., 2023). The incidence and magnitude of heavy metal toxicities vary depending on the geographical location, soil content, human activities, social customs, types and location of industries, regulatory measures for containment of pollution, healthcare facilities for detection and toxicity intervention as well as nutritional status and genetics of local population (Tchounwou et al., 2012). The environmental pollution is particularly high in point source areas including mining, foundries, smelters, and other metal-related industrial operations (Bradl, 2005; He et al., 2005). Environmental contamination may also occur through other causes or activities such as metal corrosion, soil erosion of metal ions and leaching of heavy metals, atmospheric deposition, sediment resuspension and metal evaporation from water resources to soil and ground water (Nriagu, 1989). The World Health Organization (WHO) has listed 10 major pollutants, 4 of which include heavy metals (Rehman et al., 2018). Epidemiological studies have demonstrated the association between heavy metal exposure and chronic disorders such as diabetes, respiratory diseases, renal disease, neurodegenerative disorders, cutaneous ailments, cardiovascular disease, and cancer (Kim et al., 2015; Yang et al., 2015).

Such heavy metals include the lead (Liu et al.), arsenic (As), mercury (Hg), cadmium (Cd), copper (Cu), manganese (Rafati Rahimzadeh, Rafati Rahimzadeh, Kazemi, and Moghadamnia), chromium (Cr), nickel (Ni), and aluminum (Al); all of which are highly toxic when inhaled, ingested, or experimentally inoculated in living systems. Following entry into the body, these heavy metals damage the cellular organelles and inhibit biochemical and metabolic pathways to impair physiological functions of the organs. The heavy metals have specific mechanisms of toxicity in the living systems and most cause oxidative stress which leads to an excessive accumulation of highly toxic reactive oxygen species (ROS) and free radical-associated damage to the critical organs, tissues, cells, and macromolecules (Kettunen et al.). ROS can irreversibly damage the major organs like liver, kidney, brain, heart, lung, as well as the reproductive and immune systems. Heavy metals can also cause gene mutations, carcinogenesis, skin irritation, cell damage, and cell death. The metabolism of carbohydrates, lipids, and amino acids is also affected by heavy metals. Prolonged exposures to heavy metals cause altered gene and protein expression as well as disruption of metabolism and metabolites. The damages caused by heavy metals can become life threatening and irreversible (Jaishankar et al., 2014; Briffa et al., 2020; Balali-Mood et al., 2021; Mitra et al., 2022). The major sources of heavy metals’ exposure and associated health hazards are listed in Table 1.

**TABLE 1:** Heavy metals, permissible limits, sources of exposure, and related health hazards.

Heavy metals	Permissible level (mg/L)	Sources of exposure	Health hazards	Ref
Pb	0.1	Pipes, Paints, Batteries, Sinkers in fishing, Ceramics, Paper dying, Automobile emission, Coal burning, Mining, Petrochemicals, Smoking, Manufacturing of lead-acid batteries	Effects on blood-brain barrier, Reproductive and neurological defects, Bladder and lung cancer, CVD, Hemolytic anemia	Wani et al. (2015), Rehman et al. (2018)
As	0.02	Wood preservatives, Herbicides, Agricultural pesticides, Tobacco smoke, Fungicides, Metal smelters, Contaminated water, Industrial waste	Gastrointestinal disturbances, Diarrhea, Cardiovascular disease risk, Severe vomiting, Hypertension, Diabetes mellitus, Carotid atherosclerosis, and Malignancies such as lung cancer	Chen et al. (2011), Hughes et al. (2011), Hubaux et al. (2013), Rehman et al. (2018)
Cd	0.06	Used as a stabilizer in several alloys, color pigments, and PVC products, Phosphate fertilizer, Cigarette smoking, Crops and vegetables grown in cadmium-contaminated soil due to sludge and phosphate fertilizer, Drinking water, Photovoltaic device in TV screens, Nuclear and coal power plants, Toys, Welding, Electroplating, and nuclear fission plants	Oxidative stress, Damage to the respiratory system, Neurotoxicity, Nephrotoxicity, Carcinogenic effects, Bone demineralization, and Diabetes mellitus	Rafati Rahimzadeh et al. (2017), Rehman et al. (2018)
Hg	0.01	Fluorescent light bulbs, Electrical switches, Incineration of municipal waste, Mercury bulbs, Coal power plants, Pesticides, Cosmetics, Batteries, Paper industry	Damage to the kidney, brain, and vision, Skin burns, and Nervous disorders	Rehman et al. (2018), Zulaikhah et al. (2020)
Cu	0.1	Pesticide production, Chemical industry, Mining, and Metal piping	Liver and kidney damage, Anemia, and GI tract irritation	Rehman et al. (2018), Rehman et al. (2019)

Abbreviations: Pb: Lead; As: Arsenic; Cd: Cadmium; Hg: Mercury; Cu: Copper; CVD: Cardio-vascular disease; PVC: polyvinyl chloride; GI: gastrointestinal.

## 2 Metabolomics workflow

Typically, a biospecimen such as an organ, tissue biopsy, or cultured cells from an organism is metabolically quenched using nitrogen gas. To prepare a biospecimen containing thousands of metabolites, the sample is homogenized by an electric homogenizer to obtain the liquid mixture of the sample. Since the tissue extraction process is somewhat complex, it is rather more convenient to collect the biofluids such as cell culture supernatants, sweat, breast milk, bile, feces, cerebrospinal fluid, urine, saliva, and blood. Certain metabolites can be found in both blood and urine samples, while others may be more specific to each sample type. In contrast, saliva samples generally contain a limited number of metabolites compared to blood and urine. The selection of the appropriate sample for metabolomics studies is of utmost importance, as it significantly influences the outcomes. Therefore, it is essential to handle, store, and process the samples under specific conditions to ensure the accuracy and reliability of the results. Any deviation from these specified conditions can impact the identification of metabolites and their associated pathways. Once the sample is prepared, it can be injected into one or more analytical instruments such as gas chromatography/mass spectrometry (GC-MS), NMR, liquid chromatography/mass spectrometry (LC-MS), capillary electrophoresis/mass spectrometry (CE-MS), or ion mobility spectrometer/mass spectrometer (IMS-MS) systems.

**FIGURE 1:**
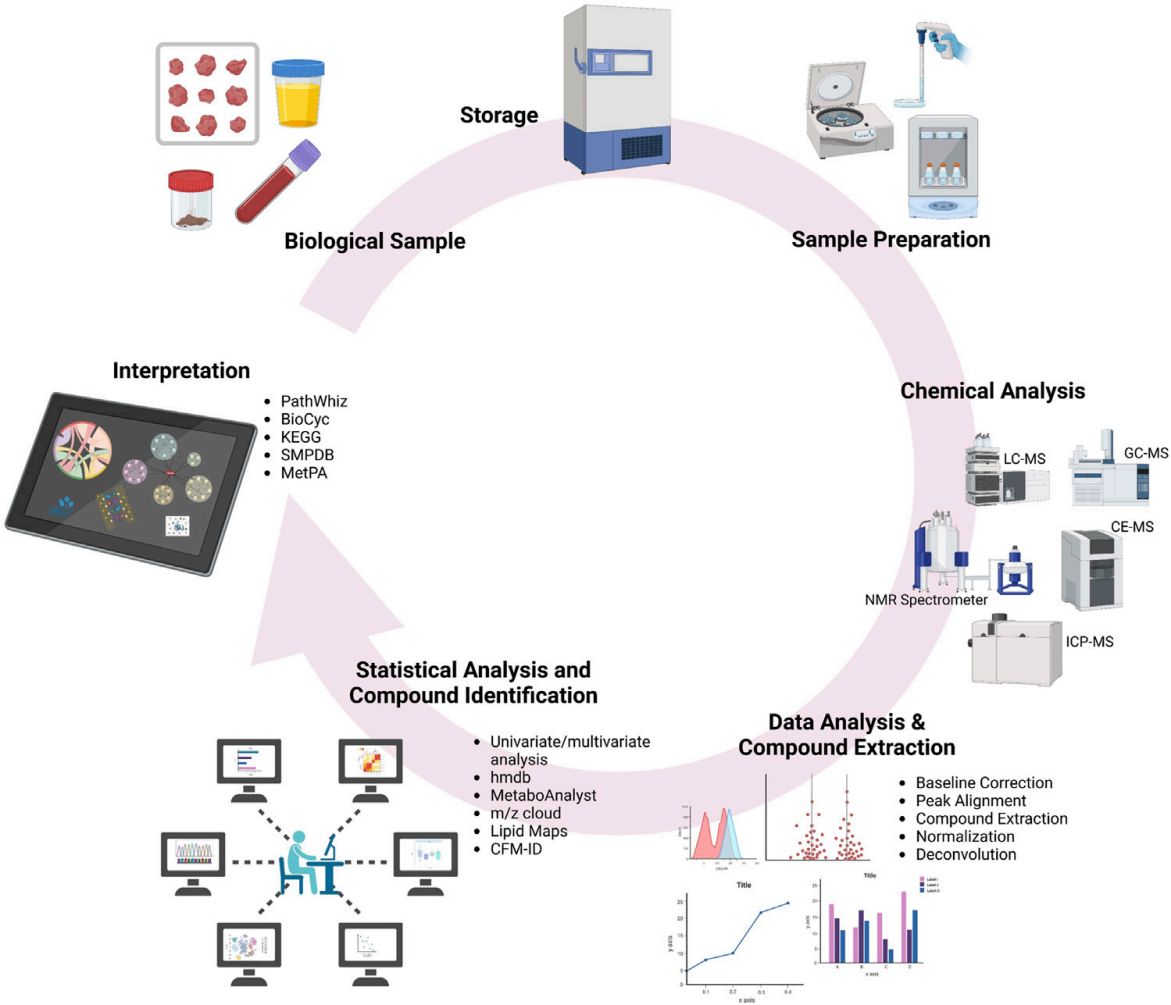
The workflow diagram of metabolomics.

Overall, to develop better workflows in heavy metal toxicity assessments using metabolomics, it is imperative to increase the reproducibility and translatability of toxicometabolomics data through the standardized approach or procedures based on appropriate selection of experimental design, optimal sample collection, storage, extraction and preparation, correct choice of analytical instruments/platform and proper data analysis, all of which can considerably affect the obtained results. Since the optimal and simultaneous extraction, detection, and quantification of all metabolites is unlikely while using a single method, use of multiple analytical platforms enhances the metabolite coverage. To date, NMR and MS are the commonest approaches used to generate metabolomics data (Araújo et al., 2021). It should also be noted that the use of *in vitro* models (primary cells, cell lines, co-cultures, 3D cultures, organ-on-chip culture, etc.) as well as using small cohorts might contribute as potential sources of bias and data variability. Furthermore, the preliminary metabolomics data obtained must be further validated, for which, putative biomarkers that have been identified using metabolomics must be accurately and precisely assessed in larger study cohorts. Special consideration should also be given to the data stored in databases and biobanks which, in case of heavy metal toxicometabolomics, still need to be further expanded and improved. A complete workflow of metabolomics is illustrated in Figure 1.

## 3 Role of metabolomics in deciphering impairment caused by heavy metal toxicities

Heavy metals have a specific density of ˃5 g/cm^3^ (Järup, 2003) and a potential to adversely affect the environment as significant pollutants and prove noxious to living organisms when the levels exceed certain permissible thresholds. At very low, normal or at biologically relevant concentrations, these metals are quintessential for the homeostatic maintenance of diverse biochemical and physiological functions in living organisms. The most common heavy metal toxicants found in the environment include the As, Pb, Cd, Cr, Hg, Cu, and Al, all of which pose dire risks for human and animal health as well as for the environment (Tchounwou et al., 2012). The common sources of heavy metal toxicants include sewage discharge, soil erosion, urban runoff, industrial and mining operations and effluents, natural weathering of earth’s crust, pesticides and other disease control agents applied to crops, etc. (Oosthuizen, 2012). Mechanistically, most heavy metals have the ability to bind with proteins at critical sites by displacing original metals from their natural binding sites, thus leading to cellular dysfunction and toxicity or by binding to DNA and nuclear proteins which results in oxidative deterioration of the biological macromolecules (Flora et al., 2008).

“As” causes serious toxicities in humans and animals and exposures commonly occur via air, food, and water, leading to organ toxicity, hypertension, cardiometabolic disease, type-2 diabetes (T2D), neurological issues, and cancers of the lung, liver, bladder and skin (Smith et al., 2000). Regarding the effect of inorganic As in diabetic and non-diabetic individuals, Martin et al. found that metabolite alterations were associated with amino acid metabolism and TCA cycle and were related to oxidative stress and endocrine disturbances as potential indicators (Martin et al., 2015). A study in male Sprague-Dawley (SD) rats also reported changes in amino acid and lipid metabolism, leading to organ toxicity, metabolic disruption and altered gene expression (Wang et al., 2015). Similarly, Zhang and Shen et al. identified that the top 5 urinary biomarker metabolites were strongly associated with endocrine disturbances and oxidative stress, concluding that metabolomics was highly useful for identifying urinary biomarkers of As-associated toxicity (Zhang et al., 2014).

Pb is used in several industries, agriculture, and home items. Common toxicities occur via inhalation and ingestion. It induces expression of free radicals and increased ROS lead to changes in lipid metabolism, DNA damage, and dysregulated gene expression, also interrupting the heme pathway to cause anemia (Rehman et al., 2018). A Pb toxicity study of inhabitants living near the used lead acid battery (ULAB) recycling plants reported urinary metabolites that were associated with impairment in hemoglobin biosynthesis pathway, small-molecule transport, nephrotoxicity, and neurotoxicity (Eguchi et al., 2018). Kelly et al. investigated Pb levels in toenails and blood, with signature metabolites in the plasma and concluded that metabolomics was a promising tool for analyzing pathogenic mechanisms of Pb toxicity including oxidative stress and immune system dysfunction (Kelly et al., 2020). In a recent study, a group of 25 albino Wistar rats was divided into different groups and treated with lead acetate (PbAc). The aim was to investigate the toxic effects of lead exposure on metabolic pathways and evaluate the potential ameliorative effect of quercetin (Que), a known antioxidant. The metabolic study conducted in this research revealed interesting findings. Specifically, the analysis identified four lipid metabolites and six amino acids that demonstrated significant changes in response to lead exposure. These alterations in the lipid and amino acid metabolism provided evidence of impairment in these specific metabolic pathways due to the toxic effects of lead. To further explore the potential protective effect of quercetin, rats were treated with Que after PbAc exposure. The results indicated that the antioxidant properties of Que exerted an ameliorative effect on lead toxicity. This suggests that Que was able to mitigate or reduce the negative impacts of lead-induced oxidative stress on the metabolic pathways. Overall, this study confirms the toxicity of heavy metals, specifically lead, in impairing metabolic pathways through the induction of oxidative stress. The identification of altered lipid metabolites and amino acids provides the insight into the specific metabolic disturbances that are caused by lead exposure. Additionally, the study highlights the potential of quercetin as a protective agent against heavy metal toxicity, potentially through its antioxidant properties (Yaqoob et al., 2022).

Cd and chlorpyrifos (CPF) are the environmental contaminants and exposures occur via inhalation (tobacco smoking) and ingestion (dietary sources), resulting in acute and chronic intoxications. These can bind with cysteine, glutamate, histidine, and aspartate, causing iron deficiency and neurotoxicity. By replacing Zn^2+^ in metallothionein, they can interfere with free radical scavenging and lead to oxidative damage (Irfan et al., 2013). Regarding toxic effects of Cd on metabolism and renal function, increased levels were associated with changes in amino acid metabolism and urine creatine pathway, and the most specific biomarkers included creatine, creatinine, l-tryptophan, adenine, and uric acid (Zeng et al., 2021). Cd, CPF, or their mixture caused neuronal damage to the rat brain, and disrupted amino acid and energy metabolism (Xu et al., 2015). Metabolic profiling of volunteers identified the potential biomarkers of Cd exposure and smoking to guide future interventions for disease prevention (Ellis et al., 2012).

Cr is found in the industrial environment such as metallurgy, electroplating, chemicals, paints, pigments, tanning, wood, pulp, and paper production. Cr toxicity causes dysfunction of antioxidant enzymes such as catalase, peroxidase, and cytochrome oxidase, leading to ROS overexpression and oxidative stress which damages the DNA/RNA, lipids, and proteins (Stohs and Bagchi, 1995).

Hg is a potentially hazardous toxicant associated with acute heavy metal poisoning in humans. Methyl mercury (MeHg) is highly neurotoxic and causes free radical formation, lipid peroxidation, damage to the ribosomes, endoplasmic reticulum (ER) and mitochondria, disruption of mitochondrial membrane potential (ΔΨm) and Ca^2+^ homeostasis, and the accumulation of neurotoxic molecules including aspartate, glutamate, and serotonin which may lead to malfunctioning of nerves, brain, kidneys, and muscles (Patrick, 2002). A toxicity study of MeHg and perfluorooctanesulfonate (PFOS) in pregnant SD rats showed weight loss, and delayed behavioral and innate immune responses in newborns, anxiety in adolescents, and increased thigmotaxic or hyperactivity in young pups. The altered cortical metabolites were associated with excitatory and inhibitory neurotransmission and defective fetal development (Reardon et al., 2019).

Cu is found in high concentrations in the liver, brain, and kidneys. It binds to ceruloplasmin in the liver and is transported to the peripheral tissues. It acts as a catalytic cofactor in redox reactions of many proteins and a balance between uptake and efflux of Cu^2+^ ions is critical to cellular Cu homeostasis. Increased Cu levels lead to oxidative stress through Fenton reactions, DNA damage, reduced cell proliferation, and metabolic disruption (Oe et al., 2016). Xiao et al. found that Cu toxicity in the intestinal HT-29 human colon carcinoma cell line caused oxidative stress, apoptosis, and altered energy and lipid metabolism and mitochondrial β-oxidation (Xiao et al., 2016). A study of untargeted metabolomics in human lung epithelial A549 cells exposed to copper oxide nanoparticles (CuO NPs) identified biomarker metabolites that were associated with hypertonic and oxidative stresses and apoptosis (Boyles et al., 2016).

Al toxicities occur by ingestion of contaminated food and beverages. Using human colon carcinoma HT-29 cell line model, Yu et al. reported that Al exposure arrested the cellular growth and proliferation, with altered metabolites/pathways of pyruvate metabolism, glutathione metabolism and TCA cycle, and mechanisms associated with lipids/amino acid metabolism, apoptosis, oxidative stress, and bioenergetics (Yu et al., 2019).

In regard with the relationship between heavy metal toxicity and breast cancer progression, the top 4 heavy metals that were significantly increased in plasma of breast cancer patients included the Cd, Pb, As, and Cr, suggesting that altered lipids and metabolites influenced the breast cancer progression (Li et al., 2020). Likewise, metabolomics of urine samples of breast cancer patients were found to have elevated levels of Cd, As, and Cr, suggesting that environmental exposure to these heavy metals affected urine metabolites and correlated with breast cancer progression (Men et al., 2020). Regarding the relationship between heavy metal toxicity and T2D, a longitudinal study reported the link between exposure to heavy metals (Pb, Hg, and Cd), renal dysfunction biomarkers (RBP, NAG, and KIM-1), and plasma indicators for T2D (AAAs, BCAAs, leptin, and adiponectin) (Valcke et al., 2019). A cross-sectional metabolomics study was conducted in 2022, among the middle to older-aged Chinese adults to investigate the potential risk of developing T2D and acute coronary syndrome (ACS) associated with heavy metal toxicity. The study aimed to explore the association between plasma heavy metals and metabolites and their relationship with the identified diseases. The analysis involved the measurement of 17 plasma heavy metals and 189 metabolites. The results revealed significant associations between heavy metals and metabolites. Among the top five identified metabolites were uridine, aspartyl phenylalanine, lysophosphatidylcholines 18:2, carnitine C14:2, and free fatty acid 14:1. Further analysis using Bayesian kernel machine regression (BKMR) confirmed the positive correlations of plasma Mn, Ba, Pb, and Co with carnitine, as well as plasma Ba, Al, Zn, and As with aspartyl phenylalanine. These findings suggested that a relationship existed between heavy metal toxicity and the biosynthesis of aminoacyl-tRNA and linoleic acid metabolism pathways. Additionally, the study revealed two unique pathways, alpha-linolenic acid metabolism, and biosynthesis of unsaturated fatty acids, that showed associations with heavy metals. The study concluded that the identified metabolites provided evidence of the risk of developing T2D and ACS due to heavy metal toxicity (Lin et al., 2022a). Furthermore, Supplementary Table S1 provides a comprehensive overview of the most common heavy metal toxicities in humans, animals, and cell lines, including details such as study type, exposure source, study design, subjects, detected metabolites, sample types, analytical instruments, software, database, and statistical models used.

Collectively, these studies demonstrate the advantages of metabolomics in assessing heavy metal toxicity. Although heavy metal toxicity may not exhibit immediate phenotypic changes, it can contribute to enhance the future risk of various chronic diseases and metabolic impairments. Metabolomics studies provide valuable information on the qualitative and quantitative changes in metabolites resulting from heavy metal exposure. Since these metabolites are involved in various metabolic and biosynthetic pathways, any impairment in these pathways can contribute to the development of progressive diseases.

## 4 Key role of oxidative stress in heavy metal toxicity pathogenesis

Humans are exposed to heavy metals via the occupational or environmental exposures. Accumulation of heavy metals in the body causes suppression or imbalance of the enzymes that are involved in antioxidant defense such as catalase, superoxide dismutase, glutathione reductase (GR), and glutathione peroxidase (GPx), resulting in generation of highly reactive free radicals or ROS (superoxide O_2_^•**−**^, hydroxyl OH^•^, peroxyl RO_2_^•^ and alkoxyl RO^•^) as well as certain non-radical species (peroxynitrite ONOO^−^ and hydrogen peroxide H_2_O_2_) and mitochondrial dysfunction (Ranjbar et al., 2014; Jia et al., 2015; Sun et al., 2022). The imbalance in the generation and scavenging of free radicals leads to oxidative stress, DNA/RNA oxidation, lipid peroxidation, protein carbonylation, metabolic impairment, and hepatotoxicity in the body (Niture et al., 2021; Renu et al., 2021). These complications further lead to dysregulation of the ER (Yokouchi et al., 2007; Rana, 2020) and mitochondrial homeostasis (Branca et al., 2020), dysregulation of autophagy (Chatterjee et al., 2014; Wang et al., 2020), cell cycle arrest, DNA fragmentation and apoptosis (Hamada et al., 1997; Yedjou et al., 2015). Heavy metals cause alteration in the expression of DNA damage repair associated p53 tumor suppressor protein (Rana, 2008), downregulated expression of p21 cyclin-dependent kinase inhibitor (CKI) (Vogt and Rossman, 2001), DNA methylation (Vaiserman and Lushchak, 2021), other epigenetic alterations (Bitto et al., 2014; Ryu et al., 2015), and histone modifications; all these factors may lead to carcinogenicity (Chen et al., 2006; Ke et al., 2008; Chen et al., 2010).

Heavy metals also have deleterious effects on kidneys and are responsible for tubular proteinuria, renal glycosuria, aminoaciduria, glucosuria, phosphaturia, and Fanconi-like syndrome (Diamond and Zalups, 1998; Lentini et al., 2017; Orr and Bridges, 2017). Nephrotoxicity is mediated by elevated expression of transcription factors MAPK/NF-κB which triggers the inflammatory processes in renal tissue microenvironment (Li et al., 2021; Hassanein et al., 2022). Accumulation of heavy metals may lead to several neurological disorders including Parkinson’s disease (Montgomery, 1995; Vellingiri et al., 2022), Alzheimer’s disease (Kitazawa et al., 2009; Kabir et al., 2021), and related dementias (Bakulski et al., 2020), resulting in the loss of homeostatic regulation, neuroinflammation and neuronal apoptosis (Huat et al., 2019; Alquezar et al., 2020). The immune function is also compromised by exposure to heavy metals, leading to changes in major immune effector cells and expression of inflammatory cytokines/chemokines (Dong et al., 1998; Bonaventura et al., 2018; Hossein-Khannazer et al., 2020; Wang et al., 2021), autoimmunity (Rowley and Monestier, 2005; Anka et al., 2022), increased allergies (Büdinger and Hertl, 2000; Vas and Monestier, 2008; Riedel et al., 2021), hematological cancers (Kim et al., 2015; Chen et al., 2016; Gianì et al., 2021; Kiani et al., 2021; Vincent Salvatore and Juley, 2021), immunosuppression (Jadhav et al., 2007; Jin et al., 2020), and diseases involving opportunistic pathogens (Lokuge et al., 2004; Krueger and Wade, 2016; Eggers et al., 2018; Vats et al., 2022) and parasites (El-Hak et al., 2022).

Chronic heavy metal exposures can lead to hyperpigmentation (Granstein and Sober, 1981; Jagadeesan et al., 2021), hyperkeratosis (Huang et al., 2019), cutaneous cancers (Järup, 2003; Uddin et al., 2007; Surdu et al., 2013) as well as cardiovascular disease (CVD) associated metabolic pathologies including hypertension (Rahman et al., 1999), atherosclerosis (Hsieh et al., 2008), reduced NO availability (Simeonova and Luster, 2004), altered renin-angiotensin system (Akther et al., 2019), disrupted vascular smooth muscle Ca^2+^ signaling (Marchetti, 2013), thrombosis (Arbi et al., 2017; Ferrante et al., 2017), suppression of vasodilator (Tan et al., 2020) and overexpression of vasoconstrictor prostaglandins (Alissa and Ferns, 2011), and increased inflammatory responses (Zhang et al., 2020; Nguyen, Oh, Hoang and Kim, 2021).

**FIGURE 2:**
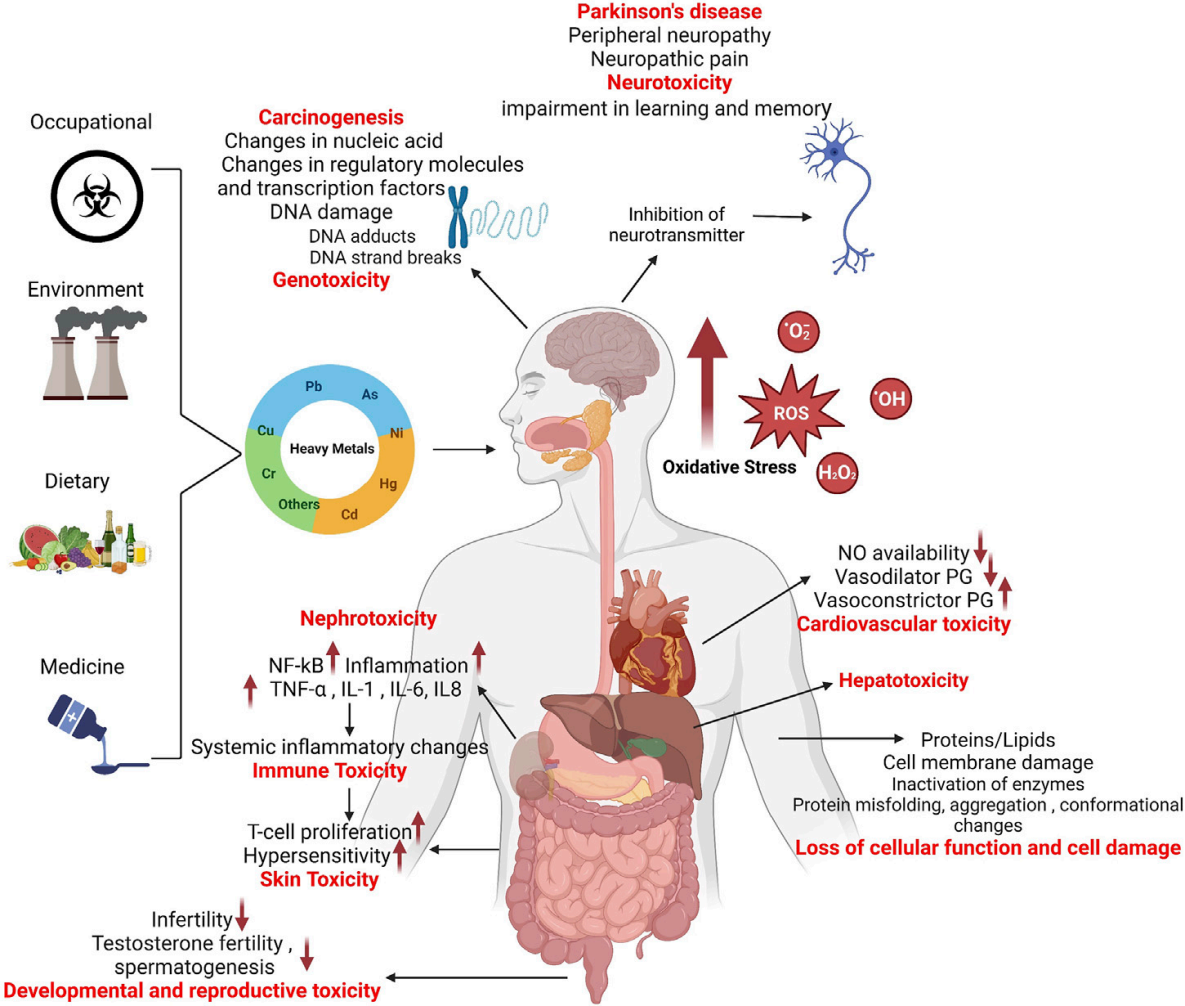
Mechanisms of heavy metal toxicity.

Overall, oxidative stress from exposure to heavy metals leads to ROS-associated metabolic reprogramming, cellular and/or organelle dysfunction, as well as perturbations of critical pathways related to carbohydrate, protein, and lipid metabolism (Wu et al., 2016). Heavy metals toxicity mechanisms are depicted in Figure 2.

## 5 Conclusion and perspectives

Compared with other relatively more established “omics”, such as transcriptomics, we have just begun to realize the robustness and enormous potentials of metabolomics as a state of the art technique to investigate heavy metal toxicities at the cellular, tissue, and organismal levels. Metabolomics provides unique perspectives on cytotoxicity-induced changes in metabolic profiles and functions. Herein, we have reviewed the studies that investigated toxicity effects of heavy metals or derivatives such as Pb, As, Cd, Cr, Hg/MeHg, Ni, PFOS, Cu/CuO NPs, and Al, using metabolomics of blood, plasma, serum, urine, toenails, brain samples of humans and animals as well as cell lines. Heavy metals cause impairment of antioxidant defense, resulting in excessive accumulation of highly reactive free radicals or ROS which emerge as key players in pathophysiology of heavy metal toxicity. Apart from causing damage to the ER and mitochondria, ROS irreversibly damage the cellular biological macromolecules including nucleic acids, proteins, and lipids. ROS-induced genotoxic changes such as abstraction or addition of bases, strand breaks in DNA sugar-phosphate backbone, and other DNA lesions may lead to cancer development. While, protein and lipid per/oxidation metabolites give rise to multiple toxicities including molecular, cell and organ damage, altered transcriptional and translational regulation, reproductive and developmental disorders, and neurological, dermatological, hepatic, renal, pulmonary, cardiovascular, hematologic, and immunological morbidities. Metabolomics yields the unique information about key metabolic changes affecting morbid processes in a wide variety of living systems by identifying, quantifying, and characterizing small metabolites with high precision. Metabolic fingerprinting and footprinting help decipher metabolites that derange homeostasis, metabolism, physiology, and immune functioning in the body.

Nonetheless, there are some challenges involved that need to be pointed out. First, while using untargeted or knowledge-discovery approach, it might be difficult to pinpoint which metabolic pathways play a key role as this type of investigations aim to measure as many metabolites as possible, given that this information provides basis of hypothesis building for future work. It is encouraging to see that better tools are becoming available for enrichment analyses and network constructions which enhances capabilities for data interpretation. Second, metabolomics might be laborious and expensive, given the chemical diversity of metabolome and rapid turnover of metabolites, both of which affect reproducibility of data generated. Third, in order for metabolomics data to be more meaningful, data integration with other systems biology approaches like transcriptomics and proteomics might be required for temporal comparison. From broader perspectives, metabolomics is a dynamic tool for cutting edge research in healthcare, pharmaceutics, food sciences, agriculture, and chemical industries. Metabolomics is guiding decision making for drug safety and biomarker discovery. In the near future, individualized metabolomics will most likely be mainstay for human health and environment monitoring and emerge as a lead analytical tool for molecular diagnostics, drug phenotyping, customized therapeutics, and much more.
