# Supplementary material for: Metabolomics: a promising tool for deciphering metabolic impairment in heavy metal toxicities
Source: Front Mol Biosci. 2023 Jul 6;10:1218497. doi: 10.3389/fmolb.2023.1218497 (PMC10357477; doi:10.3389/fmolb.2023.1218497)
Supplement: Supplementary file 1 [file Table1.docx]

**Table S1** Summary of metabolomics studies involving heavy metal exposure toxicity in humans, animals, and cell line models

| Heavy metal | Type of study | Type of exposure /Study design | No. of subjects in study | Type of equipment | Sample type | Total metabolites detected/ those involved in toxicity | Software and database used | Statistical model | Ref. |
| --- | --- | --- | --- | --- | --- | --- | --- | --- | --- |
| Pb | Human study (male only). Age >18 yr. | Environmental exposure. A cross sectional study of 44 blood and urine samples (ULAB site) and 41 control samples. A quality control sample contained an identical volume of each sample and blank was ultrapure water. For data analysis, metabolites with >40% CV were removed. | 95 | HILIC coupled with ESI-MS/MS | EDTA Plasma and Urine | 263/ N-acetyl putrescine, δ-aminolevulinic acid, ethanolamine, 1,3-diphopshateglycerat, imidazole acetic acid, 2-hydroxyglutarate, urea, s DL-pipecolic acid, sedoheptulose 1,7-bisphosphate (SBP), and glutamate | HMDB | Univariate analysis | (Eguchi et al., 2018) |
| Pb | Human study (Male only). Age 21–80 yrs. | Environmental exposure. A longitudinal, normative aging study (NAS) in men, free of known chronic disease. Self-reported information included medical/smoking histories, dietary intake, and other health-related factors. | 399 | UHPLC-MS/MS | Plasma, Serum and Toenails | 858/ N-acetyl lysine, suberate, hydroxy asparagine, sphingomyelin, pro-hydroxy-pro, tartrate, N-formyl methionine, hexanoyl glycine, vanillyl mandelate, lysine and theobromine | MetaboAnalyst 4.0. Metscape Bioinformatics tool Cytoscape | Independent linear regression models. Multivariable linear regression models | (Kelly et al., 2020) |
| Pb | Animal study (male Wister albino rats, Age 9 weeks) | Experimental exposure. Rats were divided in 5 groups. CON group received normal saline; Pb group received PbAc (120mg/kg); Pb + Q-CRN group received lead acetate followed by Que (50 mg/kg); Q-CRN group received Que; CRN group received corn oil. The study duration was 4 weeks. | 25 male Wister albino rats | ESI-MS, Biochrom Amino Acid Analyzer (AAA) | Serum and plasma | 4 lipid metabolites were qualified from serum by MS/MS including L-carnitine, sphinganine, phytosphingosine, and lysophosphatidylcholine. valine, glutamic acid, lysine, serine, asparagine, and threonine were quantitatively analyzed by AAA indication impairment in amino acid metabolism due to Pb toxicity. | ---- | One-way ANOVA | (Yaqoob, Rehman, Akash, Alvi, & Shoaib, 2022) |
| As | Human study (Female 133, Male 43). Age 18-79 yrs | Environmental exposure. A nested study profiling of metabolomic shifts in urine and plasma from 90 diabetic and 86 non-diabetic individuals, matched for As levels in drinking water, body mass index, age, and gender. Diagnosis of diabetes was based on fasting plasma glucose and oral glucose tolerance test. | 176 | GC-TOF-MS, LC-TOF-MS | Plasma and Spot Urine | 515 (221 in plasma & 294 in urine)/ Guanine, acetyl-N-formyl-5-methoxy kynurenamine (AFMK), testosterone, serine, and hippurate. Altered metabolites associated with the amino acid metabolism (glutamate, aspartate, alanine, arginine, proline, and phenylalanine) and TCA cycle | LECO's ChromaTOF software (Leco Co). Agilent Mass Hunter Qualitative Analysis Program (vB.05.00). XCMS package (v1.24.1, <http://metlin.scripps.edu>) | Multivariable regression models | (Martin et al., 2015) |
| As | Animal study (male Sprague Dawley rats). Age 6 weeks. | Experimental exposure. Animals were grouped as control (n=6) and low-dose (n=8, 0.5 ppm), medium-dose (n=8, 2 ppm), and high-dose (n=8, 10 ppm) sodium arsenite groups. Animals were treated 3 months consecutively. | 30 | UHPLC-  MS/MS | Serum | 77/ LysoPC (18:0), lysoPC (20:1), sphingosine, phytosphingosine, ceramide (d18:0/16:0), ceramide (d18:0/14:0), carnitine, palmitoylcarnitine, octadecenylcarnitine, methionine, valine, proline, tyrosine, indole acetaldehyde, pyroglutamic acid, creatine, uric acid, and cytosine. Altered metabolites related to the lipid and amino acid metabolism. | Micromass MarkerLynx applications manager Version 4.1 (Waters, UK) | Multivariate analysis | (Wang et al., 2015) |
| As | Human study (Male only). Age 19-43 yrs | Environmental exposure. A proof-of-concept study of Han ethnicity Chinese men. Both As levels and metabolomic alterations were measured in the same urine samples collected between 2008-9. Self-reported data included information on age, weight, height, education, annual family income, profession, and smoking/ alcohol consumption (past, current, never). | 127 | HPLC-qTOF-MS | Spot Urine | 61/ Testosterone, guanine, hippurate, acetyl-N-formyl-5-methoxykynurenamine, and serine. | Profile Analysis 2.0 (Bruker, USA) | Multivariate analysis | (Zhang et al., 2014) |
| Cd | Human study (Female 99 and Male 45). Age 40-75 yrs | Environmental exposure. Self -reported information from the participants included gender, age, height, weight, history of residence, occupation, health condition, and lifestyle characteristics including smoking frequency. The study participants resided in 3 nearby villages located within 200 km distance apart and had different levels of cadmium exposure but similar lifestyles. | 144 | UHPLC QqQ-MS/MS | Morning Urine and Blood | 23/imidazopyrimidines, fatty acyls, indoles, and their derivatives, carboxylic acids and their derivatives, diazines, steroid derivatives, and steroids, and keto acids and their derivatives. Cd affected urinary levels of creatinine, adenine, L-tryptophan, uric acid, and creatine. Altered metabolites affected the tryptophan metabolism, aminoacyl-tRNA biosynthesis, and purine metabolism | One-MAP/PTO software (Dalian Chemdata Solution Information Technology Co. Ltd, China). Kyoto Encyclopedia of Genes and Genomes Database (<https://www.genome.jp/keg>). Human Metabolome Database (<http://www.hmdb.ca/>) MetaboAnalyst | Multivariate statistical analysis | (Zeng et al., 2021) |
| Cd | Animal study (male Sprague-Dawley rats). Age 6 weeks | A full-factorial design experimental study involving 16 treatment groups (6 rats/group). The joint effect of Cd and chlorpyrifos (CPF) was investigated. High (1/15 LD_50_), middle (1/45 LD_50_), and low (1/135 LD_50_) doses of each chemical were used. | 96 | GC-MS | Brain samples | 40/ Five distinct brain metabolite biomarkers identified were d-gluconic acid, serine, 1,2-propanediol-1-phosphate, 2-ketoisovaleric acid, and 9H-purine | Matlab 7.1 (The MathWorks, Inc., Natick, MA, USA) | Multivariate analysis | (Xu et al., 2015) |
| Cd | Human study | Environmental exposure. Each participant provided information regarding smoking status (current, past, never), age and gender. The participants lived near the site with environmental toxicants. Data were anonymized and corrected for confounding factors including age, gender, and smoking status. | 178 | 1H NMR spectroscopy | Spot Urine | 7/ 8-oxo-deoxyguanosine, 3-hydroxyisovalerate (3-HV), citrate, and 4-deoxy-erythronic acid (4-DEA), creatinine, dimethylglycine (DMG), and creatine. | Matlab (The MathWorks, Inc., Natick, MA, USA) | Multivariate and univariate analyses | (Ellis et al., 2012) |
| Pb, As, Cr, Hg, and Cd | Human study (Female only). Age 50.22±9.83 yrs. | Environmental exposure. The malignant BC patients (n=105) and age matched healthy controls (n=35) lived in the same neighborhood with heavy metal contamination. All BC patients were at early stages (I–II), based on TNM classification. Those with diabetes, other diseases, or those that had received pre-operative treatment (adjuvant chemotherapy or radiotherapy) were excluded. All were non- smokers. | 140 | NMR, ICP-MS | Blood plasma | 40/ phenylalanine, acetate, β-glucose, citrate, propylene glycol, and leucine levels were increased, while histidine, tyrosine, α-glucose, lysine, trimethylamine, glutamate, and arginine levels were decreased in blood plasma. Levels of lipids such as VLDL (-CH_3_), LDL, HDL, lipid-C=C-CH_2_-C=C-, lipid‑CH=CH‑ and lipid-CH_2_-C=C- were also elevated | MestReC software (version 3.0; Mestrelab Research) | Multivariate analysis | (Li, Zhang, Men, Wang, & Zhang, 2020) |
| Pb, As, Cr, Hg, Cd, and Ni | Human study (Female only). Age 50.56±9.72 yrs | Environmental exposure. Patients with BC (n=106) and age-matched healthy controls (n=38) lived in the same mining area where heavy metals contamination was relatively high. All participants were non-smokers. BC patients were at early stages (I-II TNM classification). Those with diabetes, other diseases or those who received pre-operative therapy were excluded. | 144 | NMR, ICP-MS | 24 hr Urine | 26/17 small urine metabolites that were decreased included creatinine, methylhistidine, trimethylamine N oxide, 2 Ox oisocaproate, glutamine, alanine, histamine, hippurate (glycolate), taurine, malonate, dimethylamine, glutamate (N acetylamino acids), glycine, 3-hydroxyisobutyrate, valine (isoleucine and leucine), lysine, and 3-methylgutarate | MestReC (version 3.0) software | Multivariate analysis | (Men et al., 2020) |
| Cd, Pb, and Hg | Human study Male 42, Age 48-82 yrs, Female 28, Age 46–87 yrs | Environmental exposure. The participants were accessed via the Canadian Longitudinal Study on Aging (CLSA) biobank resource. | 70 | ICP-MS | Plasma and Spot Urine | 17/ renal dysfunction biomarkers including RBP, N-acetylglucosaminidase, and KIM-1; and plasma biomarkers including branched-chain amino acids (*i.e.* leucine, isoleucine, and valine), leptin, adiponectin, and aromatic amino acids (AAA) (*i.e.* tyrosine phenylalanine) were identified as risk factors for developing type-2 diabetes mellitus. Urinary mercury levels had a significant correlation with KIM-1. BCAA (valine) had a negative correlation with urinary Cd, RBP, and adiponectin. isoleucine, leucine, and valine were negatively correlated to urinary RBP. | SAS (version 9.4). Path analysis by using MplusV7 | Multiple linear regression analysis | (Valcke et al., 2019) |
| CH_3_Hg (MeHg) and PFOS | Animal study Sprague-Dawley rats (25 females and 12 males). Age 3 weeks | Experimental study. The objective was to determine if mixtures of PFOS and MeHg induce different effects than individual exposures. Also, to see if the mixture effect was different using a low- or high-dose PFOS with the same MeHg dose. At gestational day 1, pregnant dams were randomly assigned to one of five treatment groups and orally administered gelatin containing either MeHg, PFOS, combinations of both, or untreated gelatin (control). Dosing of dams continued until weaning at postnatal day 21. Offspring growth and development as well as behavioral outcomes (activity, anxiety, memory, and cognition) were examined at pre-weaning and juvenile stages. The underlying molecular mechanism were investigated by targeted metabolomics in distinct juvenile brain regions. | 37 | HPLC-MS/MS | Whole brains, Plasma, and Serum | 199/ Brain cortex concentrations of taurine, methionine, serine, GABA, glycine, proline, and T4-hydroxyproline were increased in PFSO-group, while those of serine and threonine were increased in MeHg-group. Altered cortical metabolites were related to the excitatory and inhibitory neurotransmission in rats. Toxicological interactions between MeHg and PFSO led to developmental anomalies | MetaboAnalyst 3.0 ([www.metaboanalyst.ca](http://www.metaboanalyst.ca)) | Multivariate analysis. Unsupervised hierarchal cluster analysis | (Reardon et al., 2019) |
| Cu | Human cell line study | Experimental exposure. HT-29 cell monolayers at 80–90% confluence (about 10^6^ cells per dish) were treated with CuSO_4_ (200 μM Cu) for 24h and untreated group served as control; 6 replicates each group. The quenched cells were either directly used for metabolite extraction or stored at -80 °C. | HT-29 cells | UHPLC | HT-29 human colon cancer cells | 77/Following Cu exposure, expression of *GPx*, *FAS*, *SCD 1*, *ACC*, *CDO*, *GLUD 1*, *GAMT*, and *IDH* was downregulated while that of *GR* was upregulated | Agilent Mass Hunter Qualitative software. XCMS (<http://metlin.scripps.edu/download/>). Databases HMDB (<http://www.hmdb.ca/>). METLIN (<https://metlin.scripps.edu/> ). MBrole (<http://csbg.cnb.csic.es/mbrole/>) | Univariate and multivariate analyses, Unsupervised multivariate analysis | (Xiao et al., 2016) |
| CuO NPs | Human cell line study | Experimental exposure. Human lung alveolar adenocarcinoma A549 cells (2x10^5^ cells per cm^2^ of culture dish) were exposed to 10 μg/ml CuO NPs for 0, 1, 3, 6, 12, and 24h, 1 μM STS for 0, 3, 6, 12, and 24h, and 16 μg/ml CPT for 0, 6, 9, 12, and 24 h; while the cells treated with medium only served as negative control exposure. | A549 cells | HPLC-MS, HILIC-MS | A549 human lung epithelial cells | 34/ Methyl nicotinamide was identified as a general metabolic marker for CuO NPs associated toxicity and apoptosis in A549 cells | Xcalibur 3.0.63 (Thermo Fisher Scientific), mzML data with ProteoWizard 3.0.4243 (<http://proteowizard.sourceforge.net/>), OpenMS 1.11([www.OpenMS.de](http://www.OpenMS.de))KNIME 2.11.2 (<https://www.knime.org/>), HMDB, Metlin | Univariate analysis | (Boyles et al., 2016) |
| Al | Human cell line study | Experimental exposure. Based on MTT cell viability assay, the optimum Al ion concentration used was 4 mM. HT-29 cells seeded at a cell density of 10^6^ cells/well were cultured in medium containing 4 mM Al ion and were incubated for 24h; untreated cells served as a negative control. PBS washed cells were frozen in liquid nitrogen to quench metabolism and were used for extraction. The supernatant (200 μL) was used for metabolomic analysis. | HT-29 cells | UHPLC-qTOF-MS | HT-29 human colon cancer cells | 81/ Al exposure significantly altered phosphatidylcholine, creatine, glutathione (GSH), phosphatidyl-ethanolamines metabolites, pyruvate, and TCA metabolism | Agilent Mass Hunter Qualitative software XCMS (<https://metlin.scripps.edu/>), HMDB, METLIN and MBrole database | Univariate and multivariate analyses | (Yu et al., 2019) |
| Al, Sb, As, Ba,Co,Cu),Pb,Mn,Mo,Ni,Rb, Se,Sr,Tl,,Ti,V,Zn | Human study (Male and Female, Age 63.95± 7.25 yrs) | Environmental exposure. A cross-sectional study of 1994 adults. Plasma samples were taken to determine the concentration of 23 heavy metals by ICP-MS. Serum samples were used for the identification of 189 metabolites by UHPLC-MS. | Male 918, Female 1076 | UHPLC-MS, ICP-MS | Serum and plasma | 189/12 classes, including 38 lysophosphatidylcholines and phosphatidylcholines (PC), 37 fatty acids and derivatives, 22 carnitine and acylcarnitines, 22 amino acids and derivatives, 19 lysophosphatidylethanolamines and phosphatidylethanolamines (PE), 8 bile acids, 8 phosphosphingolipids, 8 other organic acids, 4 purines and derivatives, 4 carbohydrates and conjugates, 4 benzene and derivatives, and 15 other metabolites. The significant association of metabolites with heavy metals is depicted as, 85 for Ni, 66 for Zn, 55 for V, 54 for Ba, 51 for Al, 49 for Mn, 42 for Pb, 39 for As, 37 for Ti, 36 for Se, 23 for Rb, 21 for Sr, 16 for Tl, 13 for Mo, 12 for Co, 11 for Cu, and 2 for Sb. | In-house database, MetaboAnalyst 4.0 (<https://www.metaboanalyst.ca/>) and iPath (<https://pathways.embl.de/>) | Multivariable linear regression models and BKMR model | (Lin et al., 2022) |

**Abbreviations**. Pb: Lead; As: Arsenic; Cd: Cadmium; Cr: Chromium; Hg: Mercury; Ni: Nickel; Cu: Copper; CuO: Cupric oxide; NPs: Nanoparticles; Al: Aluminum; CH_3_Hg: Methyl mercury; PFOS: Perfluoro-octane sulfonate; STS: Staurosporine; CPT: Camptothecin; MTT: 3-[4,5-dimethylthiazol-2-yl]-2,5 diphenyl tetrazolium bromide; HILIC: Hydrophilic interaction liquid chromatography; MS: Mass spectrometry; ESI-MS: Electrospray ionization mass spectrometry; HMDB: Human metabolome database; UHPLC-MS/MS: Ultrahigh performance liquid chromatography-tandem mass spectrometry; GC-TOF-MS: Gas chromatography coupled to time-of-flight mass spectrometry; TCA: Tricarboxylic acid cycle (also known as Krebs cycle); HPLC-qTOF-MS: High-performance liquid chromatography-quadrupole-time of flight mass spectrometry; UHPLC-QqQ-MS: Ultrahigh performance liquid chromatography method coupled with triple quadrupole mass spectrometry; 1H NMR: Proton nuclear magnetic resonance; ICP-MS: Inductively coupled plasma mass spectrometry; VLDL: very low-density lipoprotein; LDL: Low-density lipoprotein; HDL: High-density lipoproteins; SAS: Statistical analysis system; RBP: Retinol-binding protein; KIM-1: Kidney injury molecule-1; GABA: γ-Aminobutyric acid; GPx: Glutathione peroxidase; FAS: Fatty acid synthase; SCD-1: Stearoyl-CoA desaturase 1; ACC: Acetyl-CoA carboxylase; CDO: Cysteine dioxygenase; GLUD 1: Glutamate dehydrogenase 1; GAMT: Guanidinoacetate methyltransferase; IDH: Isocitrate dehydrogenase; GR: Glutathione reductase
